# Oral HPV infection in a clinic-based sample of Hispanic men

**DOI:** 10.1186/1472-6831-14-7

**Published:** 2014-01-24

**Authors:** Vivian Colon-López, Valerie Quiñones-Avila, Lizbeth M Del Toro-Mejías, Keysha Reyes, Manuel E Rivera, Kathleen Nieves, María M Sánchez-Vazquez, Magaly Martínez-Ferrer, Ana P Ortiz

**Affiliations:** 1 Cancer Control and Population Sciences Program, University of Puerto Rico Comprehensive Cancer Center, San Juan, Puerto Rico; 2 Department of Health Services Administration, Graduate School of Public Health, University of Puerto Rico, San Juan, Puerto Rico; 3 UPR/MDACC: Partnership for Excellence in Cancer Research Program, School of Medicine, University of Puerto Rico, San Juan, Puerto Rico; 4 Universidad Autónoma de Guadalajara, Zapopan, Mexico; 5 Río Piedras Campus, University of Puerto Rico, San Juan, Puerto Rico; 6 University of Puerto Rico Comprehensive Cancer Center, San Juan, Puerto Rico; 7 Department of Pharmaceutical Sciences, School of Pharmacy, University of Puerto Rico, San Juan, Puerto Rico; 8 Department of Biostatistics and Epidemiology, Graduate School of Public Health, University of Puerto Rico, San Juan, Puerto Rico

**Keywords:** Oral HPV infection, Epidemiology, High-risk men, STI/STD clinic, Puerto Rico

## Abstract

**Background:**

Human papillomavirus (HPV) is associated to the pathogenesis of various cancers, such as oropharyngeal squamous cell carcinoma, which has a high incidence in Puerto Rican men. Despite the burden of oral cancer in Puerto Rico, little is known about the epidemiology of oral HPV infection, particularly in high-risk men. Therefore, this study is aimed at determining the prevalence of oral HPV infection, the genotype distribution and correlates associated with oral HPV infection in men of at least 16 years of age attending a sexually transmitted infection (STI) clinic in Puerto Rico.

**Methods:**

A cross-sectional study consisting of 205 men was conducted. Participants provided a 30-second oral rinse and gargle with mouthwash. Following DNA extraction, HPV genotyping was performed in all samples using Innogenetics Line Price Assay (INNO-LiPA). A questionnaire was administered, which included a demographic, behavioral and a clinical assessment. Descriptive statistics and bivariate analysis were used to characterize the study sample. Variables that achieved statistical significance in the bivariate analysis (p < 0.05) were assessed in multivariate logistic regression models.

**Results:**

The mean age of the study sample was 38.5 ± 14.2 years. Oral HPV prevalence among men was 20.0% (95.0%CI = 14.8%-26.1%) and of HPV type 16 was 2.4% (95.0%CI = 0.8%-5.6%). Oral HPV prevalence significantly increased over increasing age categories (p-trend = 0.001). Multivariate analysis showed that oral HPV was independently associated with number of sexual partners (adjusted OR = 1.02; 95%CI = 1.01-1.03) and lifetime use of cigarettes (adjusted OR = 3.00; 95%CI = 0.98-9.16).

**Conclusions:**

Oral HPV among the sampled men in the STI clinic was high, regardless of the HIV status or sexual behavior. Interventions in STI clinics should include screening for HPV in the oral cavity for the early detection and reduction of long-term consequences of oral HPV infection, such as oropharyngeal cancer.

## Background

Human papillomavirus (HPV) infection is considered the most common sexually transmitted infection (STI) [1]. About 6 million people are diagnosed each year and approximately 9.0-13.0% of the world population (630 million people) is already infected with the disease [2]. HPV plays a role in the pathogenesis of Head and Neck Squamous Cell Carcinomas (HNSCCs) and Oropharyngeal Squamous Cell Carcinomas (OPSCCs) [3,4]. The incidence and mortality rates of HNSCCs have increased in several countries over the last three decades [5-9]. These group of cancers are clinically characterized by the presence of oncogenic high-risk (HR) HPV, predominantly type 16, that has been detected approximately in 95.0% of positive tumors [10,11]. In head and neck cancers, studies have shown that the prevalence of HPV DNA varies by cancer site and geography [12]. HPV DNA has been found in 35–50% in developed countries in contrast with the rest of the oral cavity, where HPV DNA is found in 5–15% of the cases [12]. HNSCCs have broad varying rates of incidence and mortality worldwide, with higher rates in European countries and Southeast Asia [13]. According to data from nine Surveillance, Epidemiology, and End Results (SEER) program registries from 1973 to 2004, in the United States (US), the incidence of HNSCC increased particularly in young and middle aged individuals (<60 years) and men [14]. This cancer type is currently the eighth most common cancer among men in the US [15,16].

Some studies have found several risk factors that increase the risk of HPV in the oral cavity. Tobacco smoking and alcohol consumption are the main risk factors for HNSCC [15,16]. Although the incidence of HPV-unrelated OPSCC associated with tobacco and alcohol use has declined over recent decades, OPSCC related to HPV has significantly increased [4]. These increases in the prevalence of HPV in the oral cavity may be the result of changes in sexual behaviors [10]. Oral HPV prevalence is more than 8-fold higher among sexually experienced individuals as compared to sexually inexperienced individuals and increases significantly with number of sexual partners [17]. This prevalence has been as high as 20.0% among individuals who have reported more than 20 lifetime sexual partners during their lifetime [17]. Additionally, men with multiple lifetime male sex partners (MSM) are 2 to 4 times more likely to be HPV 6 or 11, seropositive and 3 to 11 times more likely to be seropositive to HPV 16 or 18 [18]. Also, high rates of oral HPV infection have been found in HIV (Human immunodeficiency virus) seropositive individuals [1,10,19,20]. A study has also shown the HPV prevalence to be higher among current smokers (20.7%) and heavy alcohol drinkers (12.3%) [17].

Studies concerning the epidemiology of oral HPV infection in men are limited since much of the research focuses on factors associated with cervical cancer in women [21,22]. However, as the causes for the excess risk of HPV-related cancers in the US remains unclear [17], it is important to determine epidemiology the for this group. For example, although a substantial percentage of oropharyngeal cancer is attributed to alcohol (7.0-19.0%) and tobacco exposure (25.0%) [23], in the US about one quarter of the oral cancer cases in men and approximately half of those in women in Puerto Rico are not attributed to these exposures [24]. These findings for Puerto Rico are consistent with US data [25] and highlight the need to further understand the epidemiology of HPV infection in the oral cavity, as well as its relationship with oral cancers and anogenital infection.

Furthermore, the limited epidemiological and behavioral research on oropharyngeal HPV infection in men, including the high-risk groups, may delay the development of effective interventions to reduce the burden of oral HPV infection. For Puerto Rico in particular, men have had a higher incidence and mortality of oropharyngeal cancer as compared with Hispanics in the US [26]. According to the Puerto Rico Central Cancer Registry, from 2005–2009, this cancer type was considered the fourth most common cancer among men in Puerto Rico [27]. Puerto Rican men also have had a higher mortality risk of the disease as compared with non-Hispanic whites and non-Hispanic blacks in the US [26].

Therefore, given the burden of oropharyngeal cancer in Puerto Rico, the aim of this study was to (1) determinate the prevalence of HPV infection in the oral region and (2) to identify socio-demographic, behavioral and clinical risk factors associated with oral HPV infection in men attending a STI clinic in San Juan, Puerto Rico. STI clinics can be an ideal venue for targeting oral HPV infection epidemiology in high risk groups of men, such as men who have sex with men (MSM), HIV-positive men, and men with STIs other than genital warts. Therefore, data collected from this venue may offer the opportunity to reach these vulnerable and underserved populations more efficiently than general-population studies.

## Methods

### Study design and research procedures

This study consisted of a cross-sectional study with men attending a publicly funded STI clinic in San Juan, Puerto Rico. A convenience sample was drawn from 205 men from within the clinic’s waiting room, coupled with subsequent screening to confirm eligibility (at least 16 years of age) and informed consent. Study participation was voluntary and written informed consent, including Health Insurance Portability and Accountability Act (HIPAA) forms, notifying about the nature of the study, general purpose, the types of research and clinical procedures involved, were provided. All study design, written consent and data collection procedures were approved by the University of Puerto Rico-Medical Sciences Campus (UPR MSC) Institutional Review Board (IRB).

### Behavioral interview

The behavioral interview was completed in approximately 25 minutes by a trained interviewer. Domains included demographic characteristics (e.g. age, place of birth, current residence, employment, and educational attainment), detailed assessment of sexual risk (e.g. including age of onset, current sexual partners and practices), and assessment of alcohol, cigarette and drug use- (e.g. types, frequency, and mode of administration of illicit/illegal drugs such as: marihuana, cocaine, heroin, and crack). Additionally, the assessment included questions about health history (self-reported STI’s and HIV status).

### Variables of interest

A person was considered oral HPV positive if resulted positive to any of the 27 types tested, whereas all others were considered negative. Behavioral characteristics included patterns of smoking tobacco, smoking marihuana, and drinking. Lifetime and recent cigarette and marihuana smoking, as well as lifetime alcohol use were measured. Lifetime smoking was defined as having smoked at least once during the lifetime. Current smoking was defined as smoking at least one cigarette per day for the past 12 months. Years smoking was defined as the number of years spent smoking regularly. Lifetime smoking of marihuana was measured as “ever smoked marihuana”, which referred to having smoked marihuana at least once during lifetime. “Ever smoked marihuana in the past 12 months” was defined as having smoked marihuana at least once during the past 12 months. Lifetime alcohol use was measured as “ever having an alcoholic beverage”, which referred to drinking at least once during lifetime.

The self-reported sexual characteristics analyzed within the sample dealt with sexual behavior, including lifetime and recent oral sex. Men who had sex with men (MSM) was a self-reported measure from the question: “Regarding your lifetime sexual partners, how many of them have been men?” Those participants that answered “1 or more men”, were categorized as “men who had sex with men” (MSM) in their lifetime. Those that answered zero (0) were categorized as non-MSM. Lifetime oral sex was defined as having had oral sex at least once during lifetime. Recent oral sex was defined as having had oral sex at least once during the past 12 months.

### Oral HPV specimen collection

All of the specimen collection and HPV polymerase chain reaction (PCR) procedures used in this study have been extensively tested and refined, and used in multiple previously published studies [28,29]. After completion of the behavioral interview, participants underwent collection of biological specimens by trained physicians at the study site. An oral mouthwash sample was collected to obtain DNA for HPV genotyping. Oral rinse samples were collected as previously described in the National Health and Nutrition Examination Survey (NHANES) [30]. Briefly, each participant vigorously swished and gargled 10 ml of Scope mouthwash for 30 seconds. This was followed by depositing the Scope and saliva generated from the mouthwash into a specimen collection cup.

### DNA extraction/ inno-LiPA HPV genotyping testing

DNA was extracted using the DNA purification from buccal cells protocol from Gentra PureGene Kit (Qiagen, Valencia, CA) and following the manufacturer’s instructions. The concentration of each DNA was quantified and qualified spectrophotometrically using a Nanodrop HPV genotyping was determined in all study samples using the INNO-LiPA HPV Genotyping Extra assay (Innogenetics, Belgium) and following the manufacturer’s instructions. The INNO-LiPA HPV assay is a PCR-based hybridization assay that includes a cocktail of biotinylated consensus primers (small primer fragment (SPF) 10) to amplify a portion of the L1 open reading frame (ORF) of 13 established HR HPV types (16, 18, 31, 33, 35, 39, 45, 51, 52, 56, 58, 59, 66), 5 known or putative HR types (26, 53, 68, 73, and 82), 7 LR HPV types (6, 11, 40, 43, 44, 54, and 70), additional non-differentiated HPV types, and types with undefined risk (74 and 69/71). After DNA extraction, all specimens were subjected to PCR amplification (40 cycles) using the Inno-LiPA HPV Genotyping Extra Amp. The PCR product was then denatured, and a 10-μl aliquot was hybridized onto nitrocellulose strips where the HPV type-specific oligonucleotides were already bound. After 60 min at 49°C, the PCR product bound to a specific probe was detected by an alkaline phosphatase-streptavidin conjugate and colorimetric detection. Results were visually interpreted by experienced laboratory personnel using the reference guide provided. Some types are defined by a single positive probe on the genotyping strip (i.e., HPV6, -11, and -16), but others are interpreted as a combination of two to four probes (i.e., HPV18, -33, and -58). While the manufacturer provides interpretation of type detection, including “possible” types, only those that were detected unequivocally were included in the analysis.

### Statistical analysis

Frequency distributions and descriptive statistics were first used to characterize the study sample in terms of demographics, behavioral and HIV status. Overall prevalence of oral HPV infection was estimated, along with 95.0% binomial confidence interval (CI). Frequency distributions were used to describe the DNA HPV-types detected in the oral cavity. Chi-square tests, Fisher’s exact tests, and independent t-tests were used to evaluate differences in demographic, behavioral, and HIV status. Statistical significance was set at p-value <0.05. Variables that resulted significantly associated with oral HPV seroprevalence in the bivariate analyses were later evaluated for possible collinearity. A Pearson correlation of 0.5 or greater and a p-value for the two-tailed significance test <0.05 was considered as a significant correlation.

Factors significantly associated with oral HPV infection were included in a logistic regression analysis. Different binary logistic regression models were developed in order to investigate the relationship between the selected risk factors and the occurrence of any type of oral HPV infection. Associations with oral HPV prevalence were reported as crude and adjusted Odds Ratios (ORs), along with 95%CIs and significance assessed through the Wald test p-value < 0.05. Trend tests were conducted across age categories. Goodness of fit for all models was assessed using the log likelihood estimates. In order to choose the best model, the log likelihood estimates were compared by subtracting each model’s log likelihood from the base model’s log likelihood. First-order interactions between the significant factors of the chosen model were also evaluated in the logistic regression analysis.

Statistical analyses were performed using the statistical package SPSS (Version 21.0, Chicago, IL), while 95% confidence intervals (CI) for prevalence estimations were performed using the Binomial CI Calculator from STATA Statistical Software (Release 11, College Station, TX).

## Results

### Participant characteristics

The demographic and behavioral characteristics of the 205 participating men are shown in Table 1. The mean age of the study sample was 38.5 years (standard deviation [SD] = 14.2). The majority (65.2%) had a high school education or less and was employed (53.9%). Also, public health insurance was predominant among the sample (58.9%); although 18.3% reported not having any type of medical insurance.

**Table 1:** Demographic and risk related characteristics among 205 HIV+/HIV- men attending an STI clinic in Puerto Rico

**Characteristic**	**Number of subjects**	**Frequency (%)**
**Demographics**		
**Age at recruitment**		
Mean (SD) 38.5 (14.2)		
16-24	44	21.9
25-34	41	20.4
35-44	41	20.4
45-54	46	22.9
55+	29	14.4
**Level of education**		
< High School	54	26.5
High School	79	38.7
> High School	71	34.8
**Employed**		
No	43	21.1
Yes	110	53.9
**Type of health insurance**		
None	37	18.3
Public	119	58.9
Private	46	22.8
**Behavioral**		
**Lifetime smoking**		
No	54	26.5
Yes	150	73.5
**Current smoker**		
No	41	34.2
Yes	79	65.8
**Years smoking**		
Mean (SD) 23.9 (14.3)		
**Ever had an alcoholic beverage**		
No	10	4.9
Yes	194	95.1
**Ever smoked marihuana**		
No	77	37.7
Yes	127	62.3
**Ever smoked marihuana in the past 12 months**		
No	69	55.2
Yes	56	44.8
**Sexual activity**		
**MSM**		
No	136	70.5
Yes	57	29.5
**MSM who had oral sex at least once in their lifetime**		
No	5	8.8
Yes	52	91.2
**MSM who had oral sex at least once in the past 12 months**		
No	3	8.3
Yes	33	91.7
**MSW**		
No	30	15.8
Yes	160	84.2
**MSW who had oral sex at least once in their lifetime**		
No	13	8.1
Yes	147	91.9
**MSW who had oral sex at least once in the past 12 months**		
No	29	24.0
Yes	92	76.0
*Clinical*		
**HIV Status**		
No	101	49.3
Yes	103	50.2
**Years living with HIV**		
Mean (SD) 11.5 (8.4)		
**Any type of HPV**		
No	164	80.0
Yes	41	20.0
**High risk HPV**		
No	183	89.3
Yes	22	10.7
**Only high risk HPV**		
No	188	91.7
Yes	17	8.3
**Low risk HPV**		
No	190	92.7
Yes	15	7.3
**Only low risk HPV**		
No	195	95.1
Yes	10	4.9
**Multiple HPV**		
No	194	94.6
Yes	11	5.4

The mean years for smoking was 23.9 years (SD =14.3). More than half of the interviewed men had smoked at least once in their lifetime (73.5%), reported to be current smokers (65.8%), and had at least one alcoholic beverage in their lifetime (95.1%). Though the majority had smoked marihuana at least once in their lifetime (62.3%), they reported not having smoked marihuana in the past 12 months (55.2%).

Half of the sample reported having HIV (50.2%). The mean years of living with HIV was 11.5 years (SD = 8.4 years). Oral HPV infection was observed among 22.3% of the HIV positive men and 17.8% among HIV negative men. Additionally, approximately a third (29.5%) of the participants indicated having had sex with another man (MSM). The vast majority of the MSM’s interviewed in this sample had reported oral sex at least once in their lifetime (91.2%) and at least once in the past 12 months (91.7%). These results were also consistent with those who reported being heterosexual, in which a vast majority (91.9%) reported having had oral sex at least once in their lifetime and 76.0% having had oral sex at least once in the past 12 months.

### Oral HPV prevalence

The prevalence estimates for oral HPV infection by the 27 individual genotypes analyzed among the 205 participating men aged 16–81 years are shown in Table 2. The prevalence of oral HPV infection among men was 20.0% (95% CI = 14.8%, 26.1%). The prevalence of high-risk HPV infection was 10.7% (95% CI = 6.8%, 15.8%) and for low-risk HPV was 7.3% (95% CI = 4.2%, 11.8%). The most prevalent high-risk subtype was HPV-52 (2.9%), while the most prevalent low-risk subtype was HPV-6 (3.9%). HPV types 18, 26, 33, 45, 53, 59, 79/71, and 82 were not detected in this sample.

**Table 2:** Oral HPV prevalence by individual genotypes among 205 HIV+/HIV-men attending an STI clinic in Puerto Rico

**HPV type**	**n (N=205)**	**% (95% CI)**
**Any HPV type**	**41**	**20 (14.8-26.1)**
**High-Risk (HR)**	**22**	**10.7 (6.8-15.8)**
16	5	2.4
31	1	0.5
35	3	1.5
39	3	1.5
51	2	1.0
52	6	2.9
56	3	1.5
58	1	0.5
66	2	1.0
68	2	1.0
73	1	0.5
74	4	2.0
**Low-Risk (LR)**	**15**	**7.3 (4.2-11.8)**
6	8	3.9
11	1	0.5
43	1	0.5
44	4	2.0
54	1	0.5
70	1	0.5
**Multiple†**	**11**	**5.4 (2.7-9.4)**

†Those that have two or more HPV types.

### Factors associated with oral HPV infection in bivariate analysis

Table 3 shows the results for the associations between demographic, behavioral, and clinical characteristics with HPV status. Oral HPV prevalence was significantly associated with age, lifetime smoking, years smoking, lifetime marihuana use, and lifetime number of sexual partners. However, bivariate correlation analyses demonstrated that having smoked at least once during lifetime, having smoked marihuana at least once during lifetime, and years smoking were significantly correlated (p-value <0.05). Upon this result, we only included “having smoked at least once during lifetime” for subsequent analyses.

**Table 3:** Oral HPV prevalence and risk factors among 205 HIV+/HIV- men attending an STI clinic in Puerto Rico

**Characteristic**	**HPV negative% (n)**	**HPV positive% (n)**	**p-value**
**Demographic**			
**Age at recruitment**			
16-24	88.6 (39)	11.4 (5)	0.002
25-34	97.6 (40)	2.4 (1)	
35-44	73.2 (30)	26.8 (11)	
45-54	71.7 (33)	28.3 (13)	
55+	65.5 (19)	34.5 (10)	
**Level of education**			
< High School	75.9 (41)	24.1 (13)	0.154
High School	75.9 (60)	24.1 (19)	
> High School	87.3 (62)	12.7 (9)	
**Employed**			
No	79.1 (34)	20.9 (9)	0.134
Yes	82.7 (91)	17.3 (19)	
**Type of Health Insurance**			
None	83.8 (31)	16.2 (6)	0.751
Public	78.2 (93)	21.8 (26)	
Private	80.4 (37)	19.6 (9)	
**Behavioral**			
**Ever smoked**			
No	90.7 (49)	9.3 (5)	0.020
Yes	76.0 (114)	24.0 (36)	
**Current smoker**			
No	73.2 (30)	26.8 (11)	0.514
Yes	78.5 (62)	21.5 (17)	
**Years Smoking**	75.0 (87)	25.0 (29)	
Mean (SD)	22.3 (14.5)	28.7 (12.5)	0.038
**Ever had an alcoholic beverage**			
No	90.0 (9)	10.0 (1)	0.367
Yes	79.4 (154)	20.6 (40)	
**Ever smoked marihuana**			
No	87.0 (67)	13.0 (10)	0.048
Yes	75.6 (96)	24.4 (31)	
**Ever smoked marihuana in the past 12 months**			
No	69.6 (48)	30.4 (21)	0.105
Yes	82.1 (46)	17.9 (10)	
**Sexual Activity**			
**Lifetime number of sexual partners**	79.4 (158)	20.6 (41)	0.005
Mean (SD)	20.2 (28.9)	65.9 (98.2)	
**MSM**			
No	80.1 (109)	19.9 (27)	0.930
Yes	80.7 (46)	19.3 (11)	
**MSM who had oral sex at least once in their lifetime**			
No	80.0 (4)	20.0 (1)	0.673
Yes	80.8 (42)	19.2 (10)	
**MSM who had oral sex at least once in the past 12 months**			
No	33.3 (1)	66.7 (2)	0.066
Yes	87.9 (29)	12.1 (4)	
**MSW**			
No	86.7 (26)	13.3 (4)	0.525
Yes	81.9 (131)	18.1 (29)	
**MSW who had oral sex at least once in their lifetime**			
No	92.3 (12)	7.7 (1)	0.276
Yes	81.0 (119)	19.0 (28)	
**MSW who had oral sex at least once in the past 12 months**			
No	79.3 (23)	20.7 (6)	0.688
Yes	82.6 (76)	17.4 (16)	
**Clinical**			
**HIV**			
No	82.2 (83)	17.8 (18)	0.422
Yes	77.7 (80)	22.3 (23)	
**Years living with HIV**	79.2 (80)	29.8 (21)	0.644
Mean (SD)	11.3 (8.8)	12.2 (6.9)	

### Factors associated with oral HPV infection in logistic regression analyses

Results for the univariate and multivariate logistic regression models evaluating the association of demographic and behavioral variables with the likelihood of HPV infection are demonstrated in Table 4. In the unadjusted logistic regression model, factors independently associated with increased prevalence odds of HPV infection included: age, lifetime smoking and lifetime number of sexual partners. The prevalence of any type of oral HPV significantly increased over increasing age categories (p-trend = 0.001). Men who had smoked at least once in their lifetime were three times more likely to test positive for any HPV type infection (OR = 3.10; 95% CI = 1.15, 8.36) as compared with those who had never smoked in their lifetime. Also, per every unit increase in lifetime sexual partners, the prevalence of oral HPV infection increased by 2%.

**Table 4:** Logistic regression results for associations with HPV among 205 men attending an STI clinic in Puerto Rico

**Variable**	**Unadjusted OR (95% CI)**	**p-value**	**Model 1 Adjusted OR (95% CI)**	**p-value**	**Model 2 Adjusted OR (95% CI)**	**p-value**	**Model 3 Adjusted OR (95% CI)**	**p-value**
**Age at recruitment**								
16-24	1.00		1.00	0.021*	1.00	0.059*	1.00	0.074*
25-34	0.20 (0.02, 1.75)	0.144	0.15 (0.02, 1.40)	0.097	0.18 (0.02,1.62)	0.126	0.13 (0.01,1.22)	0.075
35-44	2.87 (0.90, 9.12)	0.076	2.28 (0.70, 7.43)	0.172	2.39 (0.70,8.18)	0.164	1.86 (0.54, 6.46)	0.328
45-54	3.07 (0.99, 9.52)	0.052	2.68 (0.85, 8.47)	0.093	2.77 (8.53, 8.96)	0.090	2.28 (0.69, 7.51)	0.174
55+	4.11 (1.23, 13.70)	0.022	3.68 (1.08,12.57)	0.037	2.54 (0.70,9.26)	0.157	2.15 (0.58, 7.98)	0.254
**P trend**	0.001							
***Log Likelihood***	***180.39***							
**Lifetime sex partners**	1.02 (1.01,1.03)	<0.001			1.02 (1.01, 1.03)	0.002	1.02 (1.01,1.03)	0.002
***Log Likelihood***	***180.47***				***161.22***			
**Lifetime smoking**								
No	1.00		1.00				1.00	
Yes	3.10 (1.15,8.36)	0.026	3.20 (1.14, 8.95)	0.027			3.00 (0.98,9.16)	0.054
***Log Likelihood***	***198.64***		***174.56***				***156.87***	

*overall p-value (Wald).

In the complete multivariate model (model 3), lifetime smoking and lifetime sex partners remained independently associated with detection of any type of oral HPV. Odds of detecting any type of oral HPV infection remained three-fold higher among men who had smoked at least once in their lifetime compared to those who had never smoked (OR = 3.00; 95% CI = 0.98, 9.16), after adjusting for differences in age and number of lifetime sexual partners. This association was marginally significant. Odds of detecting any type of oral HPV infection remained significantly higher among men with increased number of lifetime sex partners (OR = 1.02; 95% CI = 1.01, 1.03), after adjusting for differences in age and lifetime smoking. No significant first-order interactions were observed between age, lifetime smoking, and lifetime sex partners.

## Discussion

The prevalence of any type of oral HPV infection among men participating in this study was 20.0% (95% CI = 14.8%-26.1%). After multivariate regression analysis, any type of oral HPV prevalence was significantly higher among men with a higher mean number of lifetime sexual partners. A series of studies on oral HPV prevalence [31-35] have recently sampled specific populations (e.g. MSM or HIV + men) from which results of oral HPV prevalence have varied. For example, in Melbourne, Australia the prevalence of oral HPV among MSM’s attending a sexual health center was 13.0% [35]. This prevalence resulted slightly lower than that reported in similar study settings among MSM’s in Amsterdam (24.4%) [34] and among HIV-infected MSM’s in Italy (21.3%) [36]. Although comparisons with other studies are not possible due to differences in the sample, the prevalence of oral HPV among MSM in our study was 19.3%.

In our study, although a higher prevalence of oral HPV infection was observed among HIV positive men (22.3% in HIV-positive vs. 17.8% in HIV-negative), this association was not significant. Also, neither sexual behavior nor oral sexual practices were significantly associated with any type of oral HPV detection. This may be explained by the selection of a high sexual risk group of men attending a public STI clinic, which lacked enough heterogeneity with regards to some risk factors.

Prevalence of oral HPV significantly increased within increasing age categories. The peak prevalence was found among men aged 55–81 years of age (34.5%). This increasing trend could be explained by an increased oral HPV incidence with age [17], persistent and/or reactivated oral HPV infections [31] due to loss of immunity as age increases [17,37], and/or increased persistence among older individuals [17,38]. However, it is important to clarify that although a trend was found between oral HPV and age, the association between these variables did not remain statistically significant after adjusting for lifetime sex partners and lifetime smoking in the multivariate logistic regression model.

The multivariate logistic regression model for any type of oral HPV infection demonstrated an independent significant association with lifetime number of sex partners and a marginal independent association with lifetime smoking, after adjusting for differences in age categories. Increased odds of any type of oral HPV infection among men with increased number of sex partners is supported by the literature [17], indicating that oral HPV infection is higher among sexually experienced individuals than by nonsexual contact. The association with lifetime smoking, though marginal, may confirm the biological plausibility associated with oral HPV infection [33]. Cigarette smoking inhibits a variety of immune responses in the oral cavity [39], which may allow for increased HPV persistence [33]. The oral epithelial abrasion due to the direct exposure to the carcinogens in tobacco may also represent the pathway for increased HPV detection among smokers [35].

The findings of this study need to be interpreted with caution because of its inherent limitations. We acknowledge that perhaps the sample size of 205 men was not sufficient for this type of study. Though this number can be considered fair in other types of research, we found it to be insufficient for this type of setting, especially when creating categories for certain outcome variables and in analyses for associations with clinical characteristics.

Oral HPV infection was measured once, so it was not possible to differentiate between newly acquired, persistent, or reactivated infections [31]. Site of infection, such as the oral cavity or the oropharynx, was also not possible to differentiate, due to the oral rinse sampling technique used [31]. We recognize additional limitations in the way some of the behavioral and clinical variables were measured. This study relied upon self-reported data for HIV status, which could lead to misclassification and residual confounding. Among HIV positive men, CD4 T-cell count, viral load and highly active antiretroviral therapy (HAART) use, was not available. Also, the lack of frequency measures for alcohol, tobacco, and marihuana use limited the interpretation of the results. Perhaps measuring recent use, within the past 6 or 12 months, and measuring the quantity of use for these behavioral variables could have provided a more detailed insight on the magnitude of association between these variables and oral HPV in men. Categorizing these variables into more detailed recent use measurements could have increased validity and power.

Also, due to the small sample size among MSM’s, opportunities to determine recent MSM behavior (i.e. last 3 months) and conduct multivariable analyses were not possible. Other limitations include the lack of data regarding the use of condoms and other variables which could help us understand sexual partnering and preventive practices among MSM. Furthermore, we were unable to examine MSM sociocultural contexts, like violence, stigma, homophobia, social networks, and the construction of gender identities. Future studies need to explore these social determinants when attempting to understand the sexual behavior and HPV/HIV-related risks to which the participants of this study setting are exposed in Puerto Rico.

Since the study was conducted in a high risk STI public clinic, our findings are unlikely to be generalizable to populations at lower sexual risk. However, data collected from a highly selective population, such as a public STI clinic, may also offer an opportunity to reach targeted high-risk groups more efficiently than general population-based studies. As described in other studies [40,41], participants attending publicly funded STI clinics are likely to be young, uninsured and poor. Therefore, this scenario has helped target oral HPV infection analyses among a vulnerable population.

To our knowledge, this is the first study currently examining the epidemiology of oral HPV infection among men attending a public STI clinic in Puerto Rico. Since previous studies have reported that men in Puerto Rico have a higher incidence and mortality of oropharyngeal cancer as compared to Hispanics in the US [26], future studies should further elucidate if potential interactions between alcohol use, tobacco use, and HPV infection may explain the higher burden of oral cancer in this population. Also, preventive efforts in this area are warranted. However, primary preventive interventions in this high sexual risk setting can be too late and ineffective. Since our study demonstrated a high oral HPV prevalence, capacity building and training on malignancies in the oral cavity among the medical staff are recommended. This type of intervention in a high risk setting can be appropriate to reduce complications and comorbidities.

## Conclusions

This study reports on the prevalence of and risk factors associated with oral HPV infection in a sample of men attending a public STI clinic in Puerto Rico. Oral HPV prevalence was high, regardless of the HIV status and sexual behavior of men attending the STI clinic of interest for this study. Lifetime smoking and lifetime sex partners were independently associated with any type of oral HPV infection in the multivariate logistic regression model. Future interventions in this type of setting should focus on screening for HPV in the oral cavity as a way of early detection and reduction of malignant changes, HPV-related cancers, and other comorbidities.

## Abbreviations

HAART: Highly active antiretroviral therapy; HPV: Human papillomavirus; HIV: Human immunodeficiency virus; HIPAA: Health insurance portability and accountability act; HNSCCs: Head and neck squamous cell carcinomas; HR: High-risk; IRB: Institutional review board; Inno LiPA: Innogenetics line price assay; LR: Low-risk; MSM: Male sex partners; MSW: Men who have sex with women; NHANES: National health and nutrition examination survey; PCR: Polymerase chain reaction; PR: Puerto Rico; OPSCCs: Oropharyngeal squamous cell carcinomas; OR: Odds ratio; ORF: Open reading frame; SD: Standard deviation; SEER: Surveillance, epidemiology, and end results; SPF: Small primer fragment; SPSS: Statistical package for the social sciences; STATA: Data analysis and statistical software; STI: Sexually transmitted infection; UPR MSC: University of Puerto Rico-Medical Sciences Campus; US: United States.

## Competing interests

The authors declare that they have no competing interests.

## Authors’ contributions

VCL conceived of the study, participated in its design and coordination, and helped draft the manuscript. VQA performed the statistical analysis and drafted the manuscript. APO conceived of the study, participated in its design and coordination, and helped draft the manuscript. MS and MMF analyzed the laboratory data, participated in its design and helped draft the manuscript. LMDM, KR, MER, KN participated in data design, study logistics, coordination, and helped draft the manuscript. All authors read and approved the final manuscript.

## Pre-publication history

The pre-publication history for this paper can be accessed here:

http://www.biomedcentral.com/1472-6831/14/7/prepub
